# A study of the enablers and barriers to the collection of sociodemographic data by public health units in Ontario, Canada during the COVID-19 pandemic

**DOI:** 10.1186/s12889-024-20519-4

**Published:** 2024-11-06

**Authors:** Menna Komeiha, Gregory Kujbida, Aideen Reynolds, Ikenna Mbagwu, Laurie Dojeiji, Joseph J. O’Rourke, Shilpa Raju, Monali Varia, Helen Stylianou, Sydnee Burgess, Oluwasegun Jko Ogundele, Andrew D. Pinto

**Affiliations:** 1 Upstream Lab, MAP Centre for Urban Health Solutions, St. Michael’s Hospital, Toronto, ON Canada; 2 Peel Public Health, Mississauga, ON Canada; 3 Ottawa Public Health, Ottawa, ON Canada; 4 Department of Family and Community Medicine, St. Michael’s Hospital, Toronto, ON Canada; 5 Department of Family and Community Medicine, University of Toronto, Toronto, ON Canada; 6 Dalla Lana School of Public Health, University of Toronto, Toronto, ON Canada; 7 Upstream Lab, Li Ka Shing Knowledge Institute, MAP Centre for Urban Health Solutions, Unity Health Toronto, 30 Bond Street, Toronto, ON M5B 1W8 Canada

**Keywords:** Sociodemographic data collection, Social determinants of health, Public health, Health equity

## Abstract

**Background:**

Collection and use of sociodemographic data (SDD), including race, ethnicity and income, are foundational to understanding health inequities. Ontario’s public health units collected SDD as part of COVID-19 case management and vaccination activities. This research aimed to identify enablers and barriers to collecting SDD during COVID-19 case management and vaccination.

**Methods:**

As part of a larger mixed-method research study [1], qualitative methods were used to identify enablers and barriers to SDD collection during the COVID-19 pandemic. Purposive sampling was used to recruit participants from Ontario’s 34 public health units. Sixteen focus groups and eight interviews were conducted virtually using Zoom. Interview data were transcribed and analyzed using inductive and deductive qualitative description.

**Results:**

SDD collection enablers included: legally mandating SDD collection and having dedicated data systems, technological and legal supports, senior management championing SDD collection, establishing rapport and trust between staff and clients, and gaining insight from the experiences from local communities and other jurisdictions. Identified barriers to SDD collection included: provincial data systems being perceived as lacking user-friendliness, SDD collection “was not a priority,” time and other constraints on building staff and client rapport, and perceived discomfort with asking and answering personal SDD questions.

**Conclusion:**

A combination of provincial and local organizational strategies including supportive data systems, training, and frameworks for data collection and use, are needed to normalize and scale up SDD collection by local health units beyond the context of the COVID-19 pandemic.

**Supplementary Information:**

The online version contains supplementary material available at 10.1186/s12889-024-20519-4.

## Background

The COVID-19 pandemic exacerbated pre-existing health inequalities [2, 3], which highlighted the importance of collecting sociodemographic data (SDD) including race, ethnicity and income, to describe the impact of the virus on different populations [4]. A comprehensive understanding of the sociodemographic factors influencing the spread and impact of the virus was crucial for formulating effective public health strategies [5]. In Ontario, local public health units (PHUs) had a vital role in the collection of SDD during the pandemic.

Each PHU is governed by a Board of Health, as defined by the *Health Promotion and Protection Act* (1990) and is responsible for delivering public health programs and services to the communities they serve [6]. Part of this work includes direct client interactions, which provide an opportunity to collect SDD [7]. In Ontario’s healthcare policy environment, some of the facilitators of SDD collection include high-quality staff training and integrating data collection into existing workflows [8]. Reported barriers include lack of standardization of SDD collection tools and approaches, and concerns that asking sociodemographic questions could disrupt the therapeutic relationship [9, 10].

Missing SDD, resulting from collection barriers, can lead to the exacerbation of existing health inequities [8, 11, 12], as it paints an incomplete picture of the social needs of local populations. Recent research has emphasized a serious gap in equity-based COVID-19-related reporting standards in Canada and beyond due to the absence of equity-based metrics [13, 14]. Additionally, the lack of a uniform data collection infrastructure and the differing technological capacities of individual organizations present a challenge to routine data collection within PHUs [15].

To date, there is a gap in the literature regarding the experiences of PHU staff with collecting SDD. Available research in Canada has primarily examined the barriers and enablers to SDD collection faced by other health care organizations [11].

This study attempts to fill this literature gap by addressing the research question of: What public health unit-led practices facilitated or hindered SDD collection during COVID-19 case management and vaccine administration? The objective was to identify SDD collection enablers and barriers that PHUs can reference when working on streamlining SDD collection.

### Setting

In June 2020, the Ontario government implemented legislative changes to the *Health Protection and Promotion Act* (1990), mandating the collection of a select set of SDD by all 34 PHUs from clients who tested positive for COVID-19, and requiring this information to be reported in the Case and Contact Management (CCM) solution system [16].

The subset of SDD data in the COVID-19 CCM included official and childhood languages, racial identity, household income, and household size. During the first few months, data completeness across PHUs remained low. A report by Ontario Health and The Wellesley Institute found that, for the period of June 26, 2020, to April 21, 2021, 43% of all people diagnosed with COVID-19 (171,915/398,651) were missing data on race [17]. Varying completeness was thought to be driven in part by practices and protocol differences across PHUs, as well as case burden, with higher case burden generally coinciding with a lower data completeness rate [17].

On March 5, 2021, a subset of SDD fields was added to provincial COVaxON COVID-19 vaccination database [18]. This subset included official and childhood languages, ethnicity, racial identity, household income and household size [18]. Low data completion limited the ability of PHU staff to use individual-level SDD for vaccination planning. As a result, vaccine coverage by forward sortation area (FSA) and FSA-associated census demographic data were used to understand differences in vaccine uptake, which provided a partial understanding of underlying inequities and access issues [18].

## Methods

This study was part of a larger mixed-method research project aimed at identifying PHU-led practices that led to higher levels of data completeness during SDD collection for COVID-19 case management and vaccination [1]. Qualitative methods were employed to identify the enablers and barriers to SDD collection during the pandemic, with the goal of gaining a better and in-depth understanding of PHU staff experiences.

### Study Team

The study team included seven members who worked for PHUs and four qualitative researchers. Team members had substantial expertise in conducting qualitative research in a variety of health settings (such as public health and primary care), brought perspectives from a variety of roles in public health (such as managers and epidemiologists), and experiential knowledge from working in Ontario’s public health system.

### Study design

A qualitative descriptive approach [19–21] was utilized in the planning and design of this project. A series of virtual focus group discussions with PHU staff across Ontario were conducted. Focus groups were chosen as the primary method of data collection to allow participants to build on each other’s ideas [22] and explore common and diverging experiences. To increase participation rates and accommodate as many participants as possible, those unable to join the pre-scheduled focus group discussions were invited to participate in individual interviews. Recognizing the subjective nature of the problem, no theoretical basis was employed. This approach allowed us to capture the various experiences of participants in their own words and present the findings in a way that directly answers the initial research question.

### Sample and recruitment

Purposive sampling was employed to ensure a diverse range of perspectives from PHU staff involved in leading, planning and/or collecting SDD. Staff from the 34 PHUs in Ontario were recruited by email. Eligible participants were PHU staff members with experience and knowledge of SDD collection efforts during the COVID-19 pandemic. We purposively sought participation from four distinct peer groups: Frontline Staff, Specialist/Analyst/Epidemiologist, Middle Management, and Senior Management. Our recruitment strategy aimed to include representation from rural, urban, and mixed rural-urban PHUs. The opportunity to participate in the study was disseminated through various channels including the Association of Public Health Epidemiologists in Ontario (APHEO) and Council of Medical Officers of Health (COMOH) listservs. To increase recruitment rates, some co-authors made direct contact with PHU peers. In total, sixteen [16] focus groups (organized by peer group) and eight [8] individual in-depth interviews were conducted from February to March, 2023.

### Data collection

A semi-structured interview guide was developed with additional prompts for each question (see supplementary file). Focus groups were chosen as a data collection method to capture diverse dimensions and nuances of the topic [23]. Individual semi-structured interviews allowed for in-depth conversations and provided an opportunity to delve deeper into individual experiences [23].

To capture the unique roles and experiences of different peer groups, four variations of the interview guide were developed. The focus group sessions lasted between 45 and 80 min, while individual interviews ranged between 15 and 30 min.

Data collection was carried out by two facilitators with support from two note-takers. Each session had a single facilitator and a single note-taker. The semi-structured nature of the sessions allowed the facilitator to adapt the prompts based on individual experiences or group composition, allowing for the exploration of emerging areas of interest. Paraphrasing was used during the focus group discussions and interviews by reiterating what participants said to ensure their views were accurately represented and understood. Facilitators were deliberately chosen for their experience in conducting qualitative focus groups and were not public health professionals.

### Data analysis

All focus groups and interviews were audio recorded. Transcription was conducted by a third-party company. The transcripts were provided to the analysts as Microsoft Word documents. Data analysis utilized inductive and deductive approaches. The inductive approach allowed the research team to compare analysis within and between transcripts to identify new themes arising from the data [24]. This method captured content generated through broad questions, such as, “What do you think about PHUs collecting SDD from clients?” The deductive approach was used to analyze the data based on categories derived from previous research or in an iterative fashion using themes that emerged during the discussion and interview sessions [24]. Microsoft Excel was used to help organize the data and facilitate the analysis process.

A double coder approach was utilized whereby a primary coder identified and labeled codes on each transcript and a secondary coder reviewed them. In cases where divergent views emerged, a third coder made the final decision on any given code. Preliminary themes were developed based on the identified codes [25]. The coders then defined each overarching theme and created a final set of themes underneath which the codes were arranged in a single document [25].

## Results

Several themes were identified from analyzing the 16 focus group discussions and eight interviews, including enablers and barriers to SDD collection across Ontario’s PHUs during the COVID-19 pandemic (Table 1).

**Table 1:** Enablers and barriers to SDD collection

SDD collection enablers	SDD collection barriers
- Legally mandated SDD collection and dedicated data systems. - Senior management championed SDD collection which supported staff buy-in. - Established rapport and trust between staff and clients. - Gained insights from local communities and other jurisdictions.	- Provincial data systems perceived as lacking user-friendliness. - SDD collection “was not a priority”. - Time and other constraints on building staff and client rapport. - Perceived discomfort with asking and answering personal SDD questions.

### SDD collection enablers

#### Legally mandated SDD collection and dedicated data systems

Participants described efforts that were employed by the Ontario Ministry of Health to facilitate SDD collection by PHUs. Key steps to facilitate SDD collection during COVID-19 case management included: developing a dedicated data system (CCM) and implementing legislative amendments to mandate SDD collection. As one participant described,

Between when the pandemic was declared in March and when they came out with the new system (CCM) in August, not only had they developed a completely new system that allowed for the collection of this type of data … but they also amended the legislation to allow for the health protection and promotion act to permit the collection of this type of data as well. …That was one success there that enabled us to do that. (Participant, urban PHU, Epidemiologist-Analyst-Specialist peer group) ([1], p. 25).

#### Senior management championed SDD collection which supported staff buy-in

This theme focussed on the role of senior management (SM) in championing SDD collection, the utilization of peer-based support, and the importance of staff buy-in.

One participant stated,

[O]ur senior management, in making decisions in terms of resource allocation, would have made decisions to help support some of the technological advancements in our region to support the collection of the social determinants of health data. I see that as a way of senior management facilitating that collection … (Participant, urban PHU, Middle Management peer group) ([1], p. 25).

Participants mentioned the importance of early adopters or “champions” of SDD collection who provided peer-based support and guidance. One frontline staff member stated that: “…, you have SDD champions in some of the clinics. I think that has made a big difference in people embracing it and wanting to ask the questions.” (Participant, urban PHU, Frontline Staff peer group) ([1], p. 25).

The importance of encouraging staff buy-in and the extensive training required to explain the rationale behind SDD collection were emphasized. As one participant described: “I think it’s really important to have staff buy-in. I think it took a lot of training … in terms of being able to explain why this was done and trained the staff that was doing this work.” (Participant, urban PHU, Epidemiologist-Analyst-Specialist peer group) ([1], p. 25).

#### Establishing rapport and trust during Staff-Client Interaction

Explaining the rationale behind SDD collection and building trust played a crucial role in trust-building and creating a sense of comfort for clients. This was achieved through one-on-one conversations between frontline staff and clients, and through the involvement of community ambassadors. As highlighted by one of the interviewed SMs,

With the trust-building done beforehand where community ambassadors speak to members of the community, explain the context of the service, and some more information about the clinic, then we found clients enter that first check-in point already feeling more comfortable… We know that that did lead to higher data completeness from the trust that had been built up beforehand by community ambassadors. (Participant, urban PHU, Senior Management peer group) ([1], p. 25).

Another aspect that contributed to trust-building was providing clients with a confidential and safe environment and utilizing non-verbal collection methods to preserve client privacy. For example, some PHUs provided clients with a printed visual of the data fields, allowing them to point to their responses instead of verbally answering SDD questions. As described by one of the participants,

Sometimes related to privacy, so the client is able to point to the question if they don’t want to answer out loud, so we did provide that. Also, the client has the option if there is [sic] any questions that they don’t want to answer it’s their choice. (Participant, urban PHU, Senior Management peer group) ([1], p. 25).

#### Gained insights from local communities and other jurisdictions

Early on, valuable insights and lessons were derived from experiences in other jurisdictions and feedback from the community. This provided early evidence of the pandemic’s disproportionate impact and highlighted the significance of SDD collection. One participant emphasized the importance of information obtained from the United States,

We saw early on that we had seen information from the US that was presenting information on disparities in COVID, and we started seeing that through our outbreaks. We saw migrant workers had disproportionate COVID. We were already hearing anecdotes about race, like certain South Asian population [s], other PSWs [Peer Support Workers] who were … many of those who came from the Black community. We were hearing a lot of that anecdotally from our community partners, so we made it a priority to really understand and systematically know what was going on, particularly as [our health unit] became a hot spot with higher rates than others across the province, other health units. (Participant, urban PHU, Senior Management peer group) ([1], p. 25).

### SDD Collection barriers

#### Provincial data systems perceived as lacking user-friendliness

Lack of user-friendliness and the absence of an Indigenous identity capturing field were some of the SDD collection barriers within the provincial data systems for COVID-19 case and contact management (CCM) and vaccinations (COVaxON).

It was noted that CCM lacked user-friendliness and that locating SDD fields was challenging. As one frontline staff member described,

I always remember the early days of CCM not being very user-friendly … in terms of the questions and where they were, it was a whole different, not a module but a different section on that client’s page. So, oftentimes we would find that case investigators would miss it, if they weren’t following the procedure step by step and they didn’t have it out in front of them and they weren’t going through it as they were on the phone with the client. Because it was a separate part of the case investigation and sometimes it wasn’t being done, I think that was more in the early days where not everyone was familiar with CCM. (Participant, urban PHU, Frontline Staff peer group) ([1], p. 25).

A barrier in both CCM and COVaxON was the lack of an appropriate field to document Indigenous identity. Staff reported that this was negatively received by some clients, as it was viewed as a form of erasure and concern about underrepresentation. As described by one of the participants,

And there was a particular clinic region where there were a lot of Indigenous folks that were coming to get their vaccines, and I remember that questionnaire specifically excluded Indigenous identity under racial and ethnic group because there was insufficient time to have appropriate consultation. But it was rather negatively received in that particular context because it was seen as a form of erasure and not being present. So, while people could fill in their identity through “other”, there were a lot of questions being asked as to why Indigenous folks were excluded. (Participant, urban-rural PHU, Frontline Staff peer group) ([1], p. 25).

#### SDD collection “was not a priority”

Differential prioritization of SDD collection within PHUs, inadequate training and supports, and poor community engagement emerged as barriers to SDD collection. As described by one of the participants,

Our medical officer of health is a big advocate of it, but it was … our middle management, it was not a priority for them, and then since they were the ones who were running our pandemic response division at the time, it was not prioritized. (Participant (interview), urban PHU, Epidemiologist-Analyst-Specialist peer group) ([1], p. 25).

Another noted barrier was inadequate training and support resources for staff members, particularly for newly hired personnel. As shared by one of the participants,

There were several public health nurses that were hired, that was the last group presentation I provided … I provided organizational sociodemographic question training across the organization … there was a push that every single staff member was going to get training on how to do case and contact management. So, it was provided widely at that point, but I wouldn’t say there was recurring training as new people got onboarded. (Participant, urban-rural PHU, Frontline Staff peer group) ([1], p. 25).

Participants further noted that during surge periods, when there was an increase in the number of people diagnosed with COVID-19, some PHUs prioritized the collection of other mandatory information due to lack of capacity and time. As one participant described,

When we had high case numbers, management did say to us, here’s what absolutely should be asked during case management, and here’s what doesn’t have to be asked. And one of those that doesn’t necessarily have to be asked is the SDD. (Participant, urban-rural PHU, Frontline Staff peer group) ([1], p. 25).

Some participants spoke about how failing to engage communities early limited the ability of PHU staff to carry out SDD-related work. Participants emphasized the importance of community engagement in informing data collection, analysis, communication, and utilization. One participant described this,

In terms of barriers, not specifically the data collection, but barriers to this work overall, one challenge for us [was] trying to get some better community engagement. To help inform the data collection, the analysis, the communications, the use, because that’s… a really important piece (Participant, rural PHU, Senior Management peer group) ([1], p. 25).

#### Time and other constraints on building staff and client rapport

Participants shared that service providers often faced time constraints when attempting to establish rapport. Explaining the purpose of SDD collection and how the data will be used was time-consuming. One Middle manager acknowledged this challenge,

Our nurses did a great job at explaining why we gather that information, and how we gather that information, but it did sometimes take a great deal of their time explaining why we were gathering that information that wasn’t part of what they were actually dealing with, with the case and contact management at the time. (Participant, urban-rural PHU, Middle Management peer group) ([1], p. 25).

Clients sometimes did not have sufficient time to respond to SDD questions, particularly in situations where parents had multiple children and were required to answer the same questions for each child. One staff member highlighted this issue, stating that,

[It is] hard collecting data on children especially in vaccine clinics when people would bring you know, if they have three or four kids. You’re asking them the same question for each of the children and again, it’s the time factor and it’s the anxiety. The kids are already anxious, the parents are anxious. It’s not in a very private area. It’s loud, it’s noisy, it’s not what they’re used to. (Participant, urban-rural PHU, Frontline Staff peer group) ([1], p. 25).

The noisy environment and lack of privacy at some vaccine clinics made some clients wary of answering SDD questions. As one of the staff members described,

From my personal experience going to vaccine clinics …, the big vaccine clinic where it’s very loud, you have to shout to be heard, and you’re being asked those questions and lots of people can hear your responses. And it creates a similar situation of discomfort, I think both for the public health unit staff member who’s tasked with collecting the information, and the person who’s being asked to respond. And I think that’s probably even more so for people who are in a vulnerable population. (Participant, rural PHU, Epidemiologist-Analyst-Specialist peer group) ([1], p. 25).

#### Perceived discomfort with asking and answering personal SDD questions

Reflecting on the experiences within their respective PHUs, some participants shared that some frontline staff members felt uncomfortable about asking personal questions, fearing they might be perceived as disrespectful. This was evident when staff had to inform clients of their COVID-19 status as mentioned by one SM,

I really think the number one barrier was for the staff who had to ask the information was their feeling … The unknown that they felt and the discomfort that they felt … they were very sensitive to the clients, especially case and contact when you’re calling someone telling them that they have COVID … So, they wanted to ensure that they were being respectful, and they were being kind to the clients. I think that was the biggest barrier and challenge for them. (Participant - interview, urban PHU, Senior Management peer group) ([1], p. 25).

Participants noted that it was more challenging to elicit responses for certain SDD questions, particularly those related to household income. As one participant described: “People really don’t like the money questions… even though we know why it’s important and even when you explain that, people still do not feel comfortable answering certain questions.” (Participant, urban-rural PHU, Frontline Staff peer group) ([1], p. 25).

Participants highlighted that when staff members did not see value in collecting SDD or understand the purpose of collecting SDD, implementation was perceived as more challenging. As stated by one participant,

The challenge I keep seeing over and over is staff and manager pushback, and that is rooted in not seeing the value in it. I think it is also rooted in a lot of unconscious bias … or not even implicit bias. I have some staff who were saying, I don’t want to answer those questions, so nobody else will … but some of them are in extreme positions of privilege. I was like … the point of collecting SDD is to understand who is experiencing inequities, so you’re not experiencing the inequity. (Participant, urban PHU, Epidemiologist-Analyst-Specialist peer group) ([1], p. 25).

## Discussion

Collection, analysis and use of SDD is a key step in identifying and reducing health inequities. In 2020, driven by the urgency of addressing the COVID-19 pandemic and evidence that the pandemic was exacerbating existing health inequities, the Ontario government and all PHUs initiated and scaled SDD collection for COVID-19 cases [26–28]. Our study aimed to understand the factors that enabled or hindered SDD collection by PHUs to inform how to extend SDD collection beyond the COVID-19 pandemic. Our findings extend those reported by a related survey study that offered a descriptive picture of the reasons for low SDD data completeness in Ontario’s PHUs [1, 29]. Our qualitative findings provide a comprehensive understanding of the experiences of PHU staff and managers [1], by showing that staff members tasked with SDD collection often felt uncomfortable and did not fully understand the purpose of the task. We also describe how support from senior management played a role in enhancing staff buy-in [1]. 

To provide decision-makers with insight into areas of potential improvement, we identified challenges and opportunities for implementing SDD collection at both the local organizational, provincial, and federal levels. In considering organizational level implementation, the trust relationship during staff-client interactions emerged as both an enabler and a potential barrier to SDD collection. Results from this study showed that some staff members were reluctant to participate in SDD collection due to perceived client discomfort. These concerns are significant, as research has indicated that racialized patients, compared to their white counterparts, felt more uncomfortable providing race/ethnic information and were concerned about being subjected to discrimination [30]. This sense of discomfort was found to decrease when clients received a proper explanation of the rationale behind the collection and the privacy measures implemented to secure their data [10, 30], which highlights the importance of conducting client education activities before SDD collection.

Participants mentioned lack of training as a barrier to collecting SDD, resulting in lower rates of SDD collection [8]. Previous research has found that low compliance with SDD collection on the clients’ part was often attributed to staff’s discomfort with the collection process [31]. When staff members do not understand the complex history and current realities of racialized individuals, they may lack the tools needed to address clients’ concerns effectively [11]. Thus, incorporating cultural sensitivity training has the potential to increase staff competency with the collection process.

Obtaining demographic characteristics, such as income, occupation, and ethnicity, remains a priority for enhancing service delivery by public health agencies. Tailoring public health measures to priority populations grouped by major social determinants of health—such as age, occupation, migration status, income, and housing conditions— has proven effective in addressing community-specific needs [32] [32]. However, it is essential to acknowledge potential unintended consequences of such measures, which underscores the critical need for accurate data in decision-making processes.

The privacy-lacking environment at some vaccine clinics made clients wary of answering SDD questions. This lack of privacy made effective communication challenging [33]. Clients’ trust in the confidentiality of their interactions with healthcare staff is crucial for comprehensive data collection [34, 35]. Therefore, clinical spaces should be designed to respect clients’ privacy [36]. While acknowledging the challenges imposed by the pandemic, including the limited private space in some vaccine clinics, it remains possible to implement privacy-enhancing mechanisms [37], such as utilizing self-administered surveys [38] or Client Answer Guides that clients can point to instead of vocalizing their answers [37].

At a systems level, provincial data infrastructure also emerged in our study findings as an enabler or as a potential barrier, depending on the availability of support. Previous research has reported that the implementation of e-health systems is often hindered by staff perceptions of these systems as disruptive to the workflow [39] and time-consuming [40]. Conversely, ease of use has been identified as a key facilitator [40]. This highlights the importance of developing data capture software that is user-friendly and can be seamlessly integrated into routine workflows, which is essential for adoption in busy environments or during periods of high volume [40].

As PHUs consider SDD collection beyond the COVID-19 context, it is important to recognize that SDD collection during the pandemic in the provincial CCM and COVaxON systems only represented a subset of sociodemographic factors and lacked certain fields (e.g., immigration) that may be valuable for learning about other related health outcomes. Provincial health system partners need to adopt a core set of sociodemographic variables and develop clear mandates for SDD collection and use, supported by associated policies and frameworks [13]. This approach has the potential to advance collective provincial health equity goals and design public health interventions that better address the needs of local populations and equity-deserving groups.

Provincial health system partners should aim to provide PHUs with the resources needed to ensure proper implementation of SDD collection practices. This includes offering guidance and frameworks for data analysis and community engagement. At the PHU level, routine SDD collection will require engaging local communities in planning for SDD collection and use, as well as creating indicators and collection targets that can serve as accountability mechanisms.

The pandemic had a detrimental effect on increasing the workload [41] of healthcare professionals and imposed heightened time restraints [42]. Therefore, it is imperative to provide PHU staff with an environment that supports SDD collection as part of their workflow rather than an additional task.

We acknowledge that the results of this study are limited to the opinions and experiences shared by the participants. While we aimed to ensure representation across all PHUs, not all PHUs in Ontario participated. Nevertheless, this study captures a range of views from rural, urban, and mixed settings in Ontario. It is also noted that due to the design of the focus groups, we could not further distinguish enablers for PHUs with the highest completion rates, or barriers for those with the lowest completion rates. Finally, because PHU staff collected only select sociodemographic variables for COVID-19 cases and/or immunization, the experiences captured might not reflect challenges associated with collecting a more comprehensive suite of SDD.

Although this study is based on experiences of health workers within Ontario’s PHUs, similar barriers have been reported in research conducted across Canada. These barriers include the perceived relevance of sociodemographic questions, staff capacity, comfort with data collection, and the adequacy of resources [11]. This suggests that progress in addressing these barriers in SDD collection has been limited. To promote consistency and standardization across the entire Canadian health system, we recommend conducting further research using a systematic review approach. Additionally, future research should seek input from patients and healthcare service users regarding the barriers that discourage their participation in SDD collection. Input from service users will help identify and address current barriers, enabling the design of user- and patient-centered interventions to improve SDD collection beyond the COVID-19 pandemic.

## Conclusion

This study provided insights into the enablers and barriers affecting the collection of SDD by PHUs in Ontario, Canada, during the COVID-19 pandemic. To normalize and scale-up SDD collection for health outcomes beyond COVID-19, a combination of provincial and local organizational-level strategies are needed. These strategies should include supportive data systems, comprehensive training, and clear policies and implementation frameworks for data collection and use.

## Electronic Supplementary Material

Below is the link to the electronic supplementary material.

Supplementary Material 1: **Table 2**: Public Health Unit Peer Grouping Taxonomy

## Data Availability

No datasets were generated or analysed during the current study.
