# Supplementary material for: A study of the enablers and barriers to the collection of sociodemographic data by public health units in Ontario, Canada during the COVID-19 pandemic
Source: BMC Public Health. 2024 Nov 6;24:3061. doi: 10.1186/s12889-024-20519-4 (PMC11539653; doi:10.1186/s12889-024-20519-4)
Supplement: Supplementary file 1 — Supplementary Material 1: Table 2: Public Health Unit Peer Grouping Taxonomy [file 12889_2024_20519_MOESM1_ESM.pdf]

Table 2: Public Health Unit Peer Grouping Taxonomy

Public Health Unit	Region	StatsCAN Peer Group	Geography
Algoma Public Health	Northeast	C	Urban-rural
Brant County Health Unit	Central West	C	Urban-rural
Chatham-Kent Public Health	Southwest	C	Urban-rural
City of Hamilton Public Health Services	Central West	B	Urban
Durham Region Health Department	Central East	B	Urban
Eastern Ontario Health Unit	Eastern	C	Urban-rural
Grey Bruce Health Unit	Southwest	D	Rural
Haldimand-Norfolk Health Unit	Central West	D	Rural
Haliburton, Kawartha, Pine Ridge District Health Unit	Central East	C	Urban-rural
Halton Region Public Health	Central West	B	Urban
Hastings Prince Edward Public Health	Eastern	C	Urban-rural
Huron Perth Public Health	Southwest	D	Rural
Kingston, Frontenac and Lennox & Addington Public Health	Eastern	C	Urban-rural
Lambton Public Health	Southwest	C	Urban-rural
Leeds, Grenville & Lanark District Health Unit	Eastern	D	Rural
Middlesex-London Health Unit	Southwest	B	Urban
Niagara Region Public Health	Central West	C	Urban-rural
North Bay Parry Sound District Health Unit	Northeast	C	Urban-rural
Northwestern Health Unit	Northwest	D	Rural

Ottawa Public Health	Eastern	B	Urban
Peel Public Health	Central East	H	Urban
Peterborough Public Health	Central East	C	Urban-rural
Porcupine Health Unit	Northeast	C	Urban-rural
Public Health Sudbury & Districts	Northeast	C	Urban-rural
Region of Waterloo Public Health and Emergency Services	Central West	B	Urban
Renfrew County and District Health Unit	Eastern	D	Rural
Simcoe Muskoka District Health Unit	Central East	D	Rural
Southwestern Public Health Oxford Elgin St. Thomas	Southwest	D	Rural
Thunder Bay District Health Unit	Northwest	C	Urban-rural
Timiskaming Health Unit	Northeast	C	Urban-rural
Toronto Public Health	Toronto	G	Urban
Wellington-Dufferin-Guelph Public Health	Central West	D	Rural
Windsor-Essex County Health Unit	Southwest	B	Urban
York Region Public Health	Central East	H	Urban
